# Robust Generation of Cardiomyocytes from Human iPS Cells Requires Precise Modulation of BMP and WNT Signaling

**DOI:** 10.1007/s12015-014-9564-6

**Published:** 2014-11-13

**Authors:** Asifiqbal Kadari, SubbaRao Mekala, Nicole Wagner, Daniela Malan, Jessica Köth, Katharina Doll, Laura Stappert, Daniela Eckert, Michael Peitz, Jan Matthes, Philipp Sasse, Stefan Herzig, Oliver Brüstle, Süleyman Ergün, Frank Edenhofer

**Affiliations:** 1 Stem Cell and Regenerative Medicine Group, Institute of Anatomy and Cell Biology, University of Würzburg, 97070 Würzburg, Germany; 2 Institute of Reconstructive Neurobiologyy, University of Bonn-Life & Brain Center, 53127 Bonn, Germany; 3 Stem Cell Engineering Group, Institute of Reconstructive Neurobiology, University of Bonn-Life & Brain Center, 53127 Bonn, Germany; 4 Institute of Anatomy and Cell Biology, University of Würzburg, 97070 Würzburg, Germany; 5 Institute of Physiology I, University of Bonn-Life & Brain Center, 53127 Bonn, Germany; 6 Department of Pharmacology, University of Cologne, 50931 Cologne, Germany

**Keywords:** Human iPS cells, Cardiac differentiation, WNT signaling, BMP signaling, Lactate enrichment, Disease modeling

## Abstract

Various strategies have been published enabling cardiomyocyte differentiation of human induced pluripotent stem (iPS) cells. However the complex nature of signaling pathways involved as well as line-to-line variability compromises the application of a particular protocol to robustly obtain cardiomyocytes from multiple iPS lines. Hence it is necessary to identify optimized protocols with alternative combinations of specific growth factors and small molecules to enhance the robustness of cardiac differentiation. Here we focus on systematic modulation of BMP and WNT signaling to enhance cardiac differentiation. Moreover, we improve the efficacy of cardiac differentiation by enrichment via lactate. Using our protocol we show efficient derivation of cardiomyocytes from multiple human iPS lines. In particular we demonstrate cardiomyocyte differentiation within 15 days with an efficiency of up to 95 % as judged by flow cytometry staining against cardiac troponin T. Cardiomyocytes derived were functionally validated by alpha-actinin staining, transmission electron microscopy as well as electrophysiological analysis. We expect our protocol to provide a robust basis for scale-up production of functional iPS cell-derived cardiomyocytes that can be used for cell replacement therapy and disease modeling.

**Electronic supplementary material:**

The online version of this article (doi:10.1007/s12015-014-9564-6) contains supplementary material, which is available to authorized users.

## Introduction

In spite of recent advances in medicine cardiovascular disorders remain a major cause of mortality in the world [1]. Supply with human cardiomyocytes is generally limited due to lack of donors as well as the restricted proliferation rate of adult cardiomyocytes. Thus, with respect to use human cardiomyocytes for regenerative therapies, drug toxicity studies as well as disease modeling alternative sources are highly desired. There have been many attempts in this direction using adult stem cells such as bone marrow derived stem cells (BMSCs) [2], mesenchymal stem cell (MSCs) [3], c-kit and isl-1 positive cardiac stem cells (CSCs) [4, 5]. However there is little evidence that BMSCs and MSCs differentiate into cardioymocytes after transplantation since positive effects observed using those cells are mainly due to angiogenesis and paracrine effects [6]. Although it has been shown that CSCs can be differentiated into all cardiovascular lineages in an animal model [7], in humans there have been rare studies due to lack of donors, limited in vitro amplification as well as complicated isolation procedures of the CSCs [6, 8]. Embryonic stem (ES) cells hold great promise for providing an unlimited source of cardiac cells since ES cells self-renew indefinitely in cell culture and are able to differentiate into any somatic cell type [9]. However ethical considerations associated with the use of human embryos might represent a roadblock for clinical application [10]. Major breakthrough in this field came when Yamanaka and colleagues showed that overexpression of four transcription factors namely Oct-4, Sox2, Klf-4 and c-Myc were able to transform somatic cells into induced pluripotent stem cells (iPS) [11]. iPS technology allows generation of pluripotent stem cells from any somatic cells. Not only it overcomes ethical concerns associated with ES cells but also offers the potential of autologous transplantation since patient-specific cells can be used for cellular reprogramming [11].

Numerous protocols have been published reporting the derivation of cardiomyocyte-like cells from human ES and iPS cells. Induction of differentiation by co-culture with stroma cells has been demonstrated [12] as well as the use of embryoid body (EB) based differentiation paradigms [13, 14]. It is similar to embryonic development in some respect and cells from all three germ layers are formed during the course of differentiation. However EBs have complex microenvironments and for this reason signaling pathways are difficult to modulate explaining poor efficiency of cardiac differentiation [15]. Moreover, there is a significant line-to-line variability with respect to the method of reprogramming used and iPS quality resulting in up to 100-fold differences in lineage specific gene expression amongst the lines treated with same protocols [16]. Such variability within a broad range of pluripotent cell lines greatly limits its application [17, 18]. Several approaches have been published utilizing monolayer culture of cells in a serum free condition having growth factors such as BMP4, Activin A, FGF2, VEGF in order to increase the efficiency while reducing the heterogeneity arising during EB based differentiation [19–21]. However, it has been shown that optimal concentrations of growth factors greatly vary among different iPS lines. A study by Kattman et al. as well as follow up report by Sa et al. systematically showed different requirements of Activin A and BMP4 concentration for efficient cardiomyocytes yield amongst different pluripotent cell lines [22, 23]. Thus, robust and efficient cardiac differentiation requires the optimization of the protocol for each individual line, which makes it laborious [24].

Recent advances in cell signaling studies have shed light on detailed signaling pathways involved during cardiac differentiation. It has been shown that WNT signaling plays a critical role during cardiogenesis [25]. It has been suggested that during early embryonic differentiation WNT is required for mesodermal specification, however, later on cardiac specification is hampered by WNT signaling and thus inhibition of WNT signaling might be necessary for the formation of cardiomyocytes [26]. Recent studies have shown the use of WNT inhibiting small molecules for increasing the cardiomyocyte yield in the case of EB [27] as well as monolayer-based protocols [28, 29]. Moreover, two recent studies by Cao et al. have shown importance of ascorbic acid in increasing the cardiac differentiation efficiency by affecting MEK/ERK pathway. By combining the application of BMP4, CHIR99021 and ascorbic acid they were successful in isolation of cardiovascular cells from human pluripotent stem cells [30, 31]. However, varied differentiation propensities of multiple pluripotent cell lines to a particular protocol require more alternative approaches. Hence, still it is highly desired to devise simple and efficient protocols to achieve high robustness and efficacy. Here we show improvement in cardiac differentiation efficiency using precise modulation of WNT and BMP signaling. We identify an optimal combination of concentrations of BMP4 and CHIR99021 and finally perform cardiomyocyte enrichment by lactate supplementation [32]. By that within 15 days we achieve generation of more than 90 % cardiomyocytes as judged by cardiac Tropononin T staining with unmatched low line-to-line variability.

## Materials and Methods

### Human iPSC Cultivation and Subsequent Cardiac Differentiation

Human iPSC were maintained on hESC-qualified Matrigel(TM) (BD Biosciences) coated plates in mTESR1 (STEMCELL Technologies) medium until they reached 80 to 90 % confluency. Cardiac differentiation was induced by BMP4 (Life technologies) (25 ng/ml) and CHIR99021 (5 μM) (Sigma-Aldrich) in RPMI1640 (Life technologies) medium containing B27 (Life technologies) and 2 mM glutamine (Life technologies) and 50 μg/ml L-Ascorbic acid (Cell culture tested powder; Sigma-Aldrich) as a basal medium. After 24 h cells were kept in the same basal medium with CHIR (5 μM) only for 18–36 h. Afterwards cells were kept in RPMI basal medium with B27 without insulin for 24 h then medium was replaced with similar basal medium having WNT inhibitor either 10 μM of XAV939 (Sigma-Aldrich) or IWR1 (Sigma-Aldrich) for 5 days. Afterwards cells were kept 4 to 5 days in basal medium (B27 + insulin) followed by replacement with cardiac enrichment medium (RPMI 1640 without glucose (Life technologies) + 4 mM sodium L-lactate [32] (Sigma-Aldrich). Cells were kept in enrichment medium for 4 to 5 days. After enrichment phase medium was switched back to basal medium (RPMI + B27 + glutamine).

### Cell Lines

del-AR1034ZIMA 001 and fl-AR1034ZIMA 001 are lentiviral reprogramming derived iPS sister clones from human dermal fibroblasts (del: transgene excised, fl: transgene floxed [33]. Human iPS cell line (k-hiPS) [34] is a lentivirally derived iPS cell line from human keratinocytes kindly gifted by Dr. Stefan Liebau from University of Tübingen, Germany. Human iPS cell line iLB-C-50-s9 is a Sendai virus derived iPS cell line from human cord blood cells. Human iPS cell line iLB-C1-30 m-r12 is a retroviral reprogramming derived iPS cell line from human dermal fibroblasts cells [35]. As WNT reporter cell line we used a neural stem cell line (I3 lt-NES) carrying the 7TGP WNT reporter construct developed by Fuerer & Nusse (2010) [36] and obtained via Addgene (#24305).

### Immunostaining

For cardiomyocyte characterization, immunostaining was performed using anti-cTNT (Abcam; 1:100) and anti-alpha-actinin (Sigma-Aldrich; 1:200) as primary antibodies and Alexafluor-488-cojugated anti-mouse IgG as a secondary antibody. Briefly, cells were washed with PBS (Life technologies), fixed with 4 % PFA (Sigma-Aldrich) for 15 min and permeabilized in PBS containing 0.1 % Triton X-100 (Sigma-Aldrich) and 5 % FCS (Life techologies) for 30 min. Cells were then incubated overnight with the primary antibodies. Next day, secondary antibody Alexafluor-488-cojugated anti-mouse IgG (1:1,000; Life technologies) were used to detect and visualize the primary antibodies. All antibodies were diluted in blocking solution. Similar protocol was also used for the staining using ISL1 (Biorbyt; 1:200) antibody. Micrographs were taken with an Axiovert 200 M microscope (Carl Zeiss).

### RT-PCR

Total RNA was prepared with the NucleoSpin RNA kit (Macherey -Nagel) and treated with DNase (Thermo Scientific). RNA (1 μg) was reverse transcribed into cDNA via Oligo (dT) with SuperScript III Reverse Transcriptase (Invitrogen). PCR was perfomed using Go Taq polymerase kit (Promega) with following conditions; 95 °C for 2 min; followed by 34 cycles of 94 °C for 30 s, 60 °C for 30 s, and 72 °C for 45 s; followed by a single cycle of 72 °C for 5 min using the primers [37] given below.

**Table:** 

Primers	Band size (bp)	Tm (^0^C)
Oct4-F: aac ctg gag ttt gtg cca ggg ttt	120	60
Oct4-R: tga act tca cct tcc ctc caa cca		
Tbrachyury-F: tgt ccc agg tgg ctt aca gat gaa	140	60
Tbrachyury-R: ggt gtg cca aag ttg cca ata cac		
ISL1-F: cac aag cgt ctc ggg att gtg ttt	200	60
ISL1-R: agt ggc aag tct tcc gac aa		
Nkx2.5-F: gcg att atg cag cgt gca atg agt	220	60
Nkx2.5-R: aac ata aat acg ggt ggg tgc gtg		
cTNT-F: ttc acc aaa gat ctg ctc ctc gct	160	60
cTNT-R: tta tta ctg gtg tgg agt ggg tgt gg		
β-actin (BA)-F: ttt gaa tga tga gcc ttc gtc ccc	130	60
β-actin (BA)-R: ggt ctc aag tca gtg tac agg taa gc		

### Flow Cytometry

1 × 10^6^ cells were trypsinized and fixed with 4 % PFA for 10 min. Cells were then washed with PBS, permeabilized in PBS containing 0.1 % Triton X-100 and 5 % FCS for 30 min and incubated for 2 h with cTNT antibody (Abcam; 1:100). No antibody was taken as a negative control. Cells were then washed once with PBS containing 0.1 % Tween-20 and resuspended in PBS containing 0.1 % Triton-X 100 and 5 % FCS and secondary antibody Alexafluor-488-cojugated anti-mouse IgG (1:1,000; Life technologies) for 1 h in dark. Finally, cells were washed again with PBS containing 0.1 % Tween-20 and measured for FACS analysis. Analysis was performed by Flow Jo program.

### Whole-Cell Calcium Current Measurements

Conventional whole-cell patch clamp recordings were performed at room temperature in bath solution containing (mM): NaCl 137, CsCl 5.4, CaCl_2_ 2, MgCl_2_ 1, glucose 10, HEPES 10 (pH 7.4 with NaOH). Borosilicate pipettes (2–3 MΩ) were filled with a solution containing (mM): CsCl 120, MgCl_2_ 1, Mg-ATP 4, EGTA 10, HEPES 5 (pH 7.2 with CsOH). Giga-Ohm seals were formed by gentle suction. Membrane capacitance was automatically displayed in the pClamp 10.2 software (Axon instruments). Cells were depolarized from a holding potential of −80 mV to −40 mV for 45 ms in order to inactivate sodium channels. This prepulse was followed by test voltages ranging from −40 to +50 mV in 10 mV steps (pulse duration 150 ms) with a 3 s interval between single pulses.

### Calcium Imaging

Calcium imaging was performed in cell cultures loaded with 5µM of calcium indicator dye fluo-4AM (Life technologies). Briefly, the cells were cultivated in 6 well plates and incubated then with 1 ml of loading dye solution containing fluo-4 AM at room temperature for 20 min in the dark. Movies were captured through the Keyence Microscope BZ-9000 (Keyence, Japan)

### Action Potential and Ramp Recordings

Cardiomyocytes were enzymatically dissociated at day 30 and plated at low density on glass cover slips coated with fibronectin. Action potentials and voltage ramps from −100 mV to 60 mV, 250 ms long were recorded on spontaneously beating cardiomyocytes with a EPC 10 amplifier (Heka Electronics) as previously described [38]. Electrode resistance was between 2.5 and 3.5 MΩ, the pipette solution contained (in mM): 50 KCl; 80 KAsparatate; 10 EGTA; 10 Hepes; 3 MgATP; 1 MgCl_2_ (pH 7.2) and the extracellular solution contained (in mM): 140 NaCl; 5.4 KCl; 1.8 CaCl_2_; 1 MgCl_2_; 10 Hepes; 10 Glucose (pH 7.4).

### Transmission Electron Microscopy

Undifferentiated iPS cells or differentiated iPS cells at day 21 were fixed in 4.5 % glutaraldehyde in 0.1 M phosphate buffer pH 7.2 (PB). After washing with 0.1 M PB, specimens were subsequently fixed for 1 h with 1 % osmiumtetroxide in PB and washed with water. Specimens were then dehydrated in ascending concentrations of ethanol including en-bloc contrasting using 2 % uranylacetate in 70 % ethanol for 1 h. Subsequently, they were embedded in Epon812 and used for preparation of ultrathin sections which were poststained with 2 % uranylacetate and 0, 2 % lead citrate. The sections were observed using a LEO AB 912 transmission electron microscope (Zeiss NTS, Oberkochen, Germany)

## Results

### Defining Optimal Window of WNT Modulation to Achieve Efficient Myocardial Induction in Human iPSCs

**Fig. 1:**
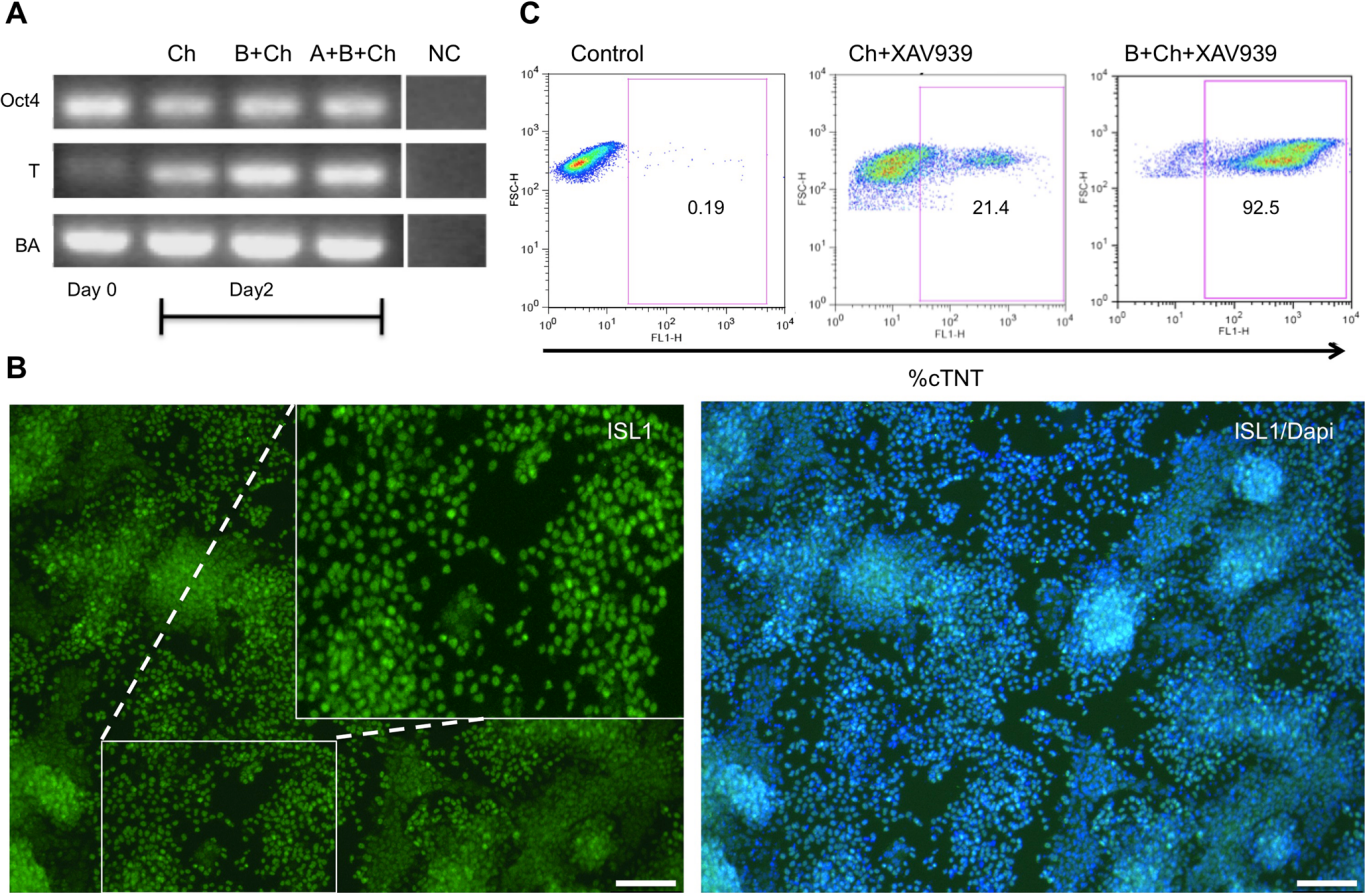
Optimization of myocardial induction of human iPS line (iLB-C-50-s9). **a** RT PCR analysis to assess the expression of T brachyury at day 2 of cardiac induction using different conditions, namely Ch, B + Ch and A + B + Ch **b** Immunostaining using cardiac precursors maker ISL1 at day 5 of cardiac differentiation using small molecule combination (B + Ch). Scale bar: 100 μM **c** Flow cytometry analysis of cardiac-specific troponin T staining at day 15 of cardiac differentiation showed 21.4 and 92.5 % of cTNT positive cells in the case of only CHIR99021/XAV939, and a combination of BMP4 with CHIR99021/XAV939, respectively. Abbreviations: *T* T-brachyury, *BA* beta-actin, *Ch* CHIR99021, *B* BMP4, *A* Activin A, *NC* negative control; *cTNT* cardiac troponin T

Since cardiomyocyte differentiation critically depends on WNT signaling, our first step was to screen molecules with which one can tightly control WNT signaling in iPS cells and their progeny, both in an agonistic and antagonistic manner, respectively. In order to quantitatively assess the level of WNT signaling, we employed a WNT reporter cell line and assessed the functionality of candidate molecules to modulate WNT signaling. In order to identify potent WNT activator, we used CHIR99021 (designated CHIR hereafter) and BIO. We found that 5 μM CHIR99021 (designated CHIR hereafter) strongly activates WNT signaling (data not shown) while BIO appeared toxic to the cells. To identify potent molecules with potent WNT inhibition, we screened previously described molecules XAV939, IWR1, KY02111 and WNT-C59. According to this analysis XAV939 and IWR1 showed strongest WNT inhibition without causing excessive cell death. Hence we decided to use CHIR as WNT activator and XAV939 or IWR1 as WNT inhibitor during the subsequent experiments. In order to achieve efficient cardiovascular induction we decided to formulate different combinations of growth factors and small molecules modulating important signaling pathways such as TGFβ (Activin A, BMP4) and FGF (FGF2) as well as WNT (CHIR, XAV939, IWR1) [15]. As a quick read out for cardiovascular induction we decided to check the expression of T-brachyury at day two (Fig. 1a) and Isl1 at day 5 (Fig. 1b) of differentiation. As an end-point analysis we monitored the capability of cultures to exhibit spontaneously beating patches in a semi-quantitative manner (Suppl. Tabl. 1-3). Comprehensive quantification of cardiac differentiation was carried out by flow cytometry analysis using cTNT specific antibodies (Fig. 1c). According to these analyses we found that the combination of BMP4 (25 ng/ml) and CHIR (5 μM) strongly enhanced expression of T-brachyury whereas additional application of Activin A had no effect (Fig. 1a). Neither increasing the CHIR concentration nor extending the incubation period beyond 48 h had a beneficial effect (Suppl. Tabl. 1). Applying 25 ng/ml BMP4 turned out to be the optimal concentration since higher and lower concentrations, respectively, reduced the number of beating patches (Suppl. Tabl. 2). After an initial phase of WNT activiation the precise timing of WNT inhibition is critical. Our data shows that application of both WNT inhibitors, XAV939 or IWR1, between day 3 and 8 results in optimal cardiac differentiation (Suppl. Tabl. 3). After day 5 of differentiation cells showed strong expression of the early cardiac marker ISL1 in almost every cell (Fig. 1b). Using these optimized conditions we generated cardiomyocyte-like cells of up to 95 % purity from human iPS line (iLB-C-50-s9) at day 12 of differentiation as judged by flow cytometry analysis using cTNT specific antibodies (Fig. 1c).

### Lactate Based Cardiac Enrichment Strongly Reduces Line-to-Line Variability of Cardiomyocyte Differentiation

**Fig. 2:**
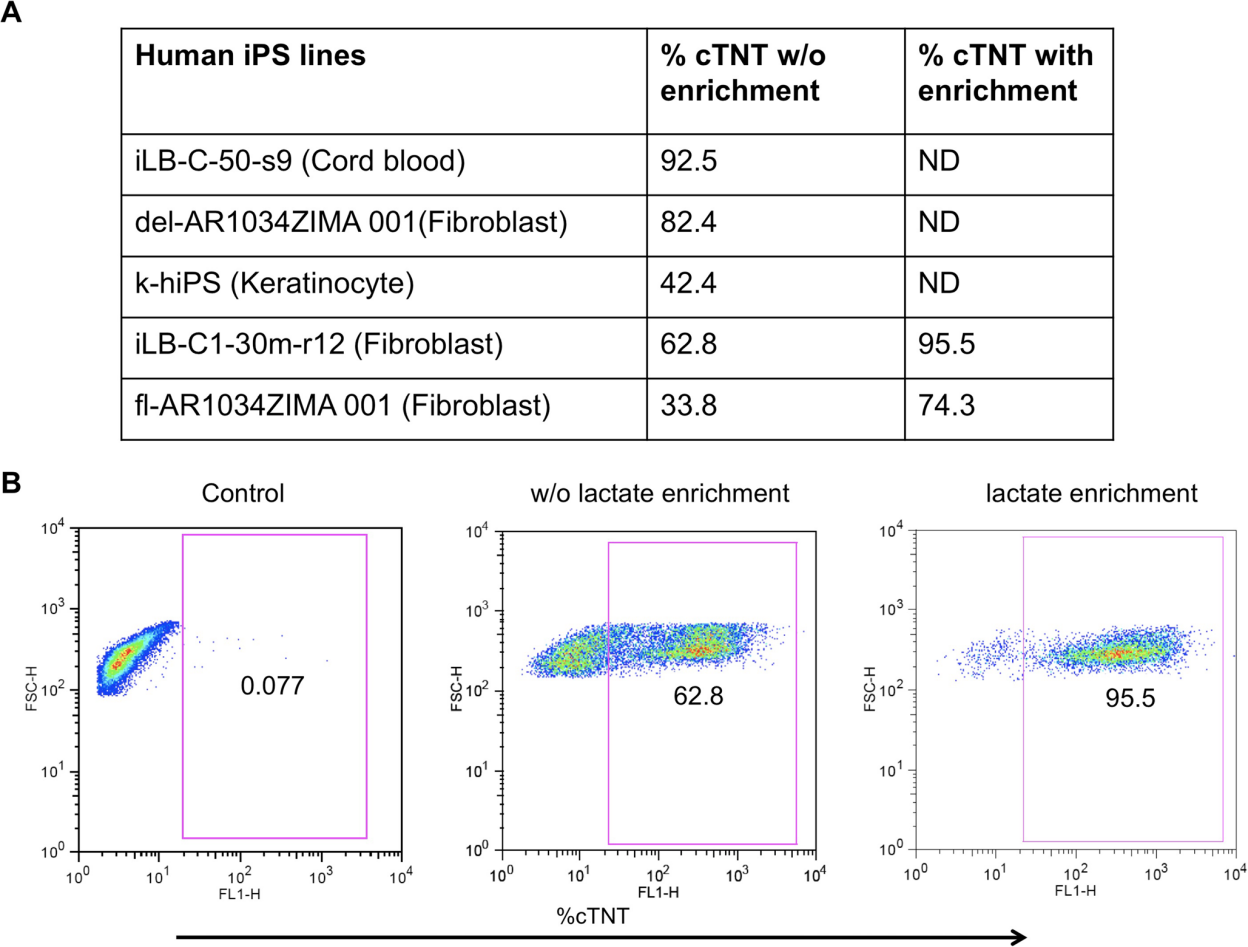
Enrichment of cardiomyocytes with sodium L-lactate. **a** Summary of cardiac differentiation of different human iPS lines using efficient cardiac differentiation followed by lactate enrichment. **b** Flow cytometry analysis of cardiac-specific troponin T staining at day 16 of cardiac differentiation of line iLB-C1-30 m-r12 showed about 63 % cTNT positive cardiomyocytes without lactate enrichment and 96 % cTNT positive cells after lactate enrichment

After optimization of cardiac differentiation using a standard iPS line, we checked the efficacy of the devised protocol on multiple iPS lines representing different origins of cells (fibroblasts, keratinocyte and cord blood cells) as well as methods of reprogramming (Retrovirus, Lentivirus and Sendai virus) to cover the full spectrum of state-of-the-art iPS technology (see details on iPS lines used in the materials section). Although our optimized protocol gave rise to a highly enriched population of beating cells with the standard iPS cell line (del-AR1034ZIMA 001), the outcome with the other iPS lines indeed varied substantially. In fact, we obtained yields of cTNT-positive cells ranging from 33 to 92 % (Fig. 2a) demonstrating the high line-to-line variability using the basic standard protocol. In order to maximize purity of cardiomyocytes from different iPS lines to the same level, we decided to apply lactate based cardiac enrichment in the late phase of our protocol. As has been recently reported glucose-depleted, lactate-supplemented culture medium strongly selects for cardiomyocytes [32]. Since only cardiomyocytes can metabolize lactate for energy supply, other non-cardiac cells were expected to die out during this 4 days treatment resulting in higher purity of cardiomyocytes. In order to achieve this, we switched the medium at day 12 of cardiac differentiation to basal medium without glucose but supplemented with lactate. In fact when we applied lactate enrichment, we could obtain 95 % pure cTNT-positive cells from the iPS line iLB-C-30-r12 which otherwise gave about 63 % positive cardiomyocytes (Fig. 2a and b). Even the iPS line fl-AR1034ZIMA, carrying loxP-flanked reprogramming transgenes [35] and being strongly resistant towards cardiac differentiation, showed efficient enrichment from 34 to 74 % cTNT-positive cells (Fig. 2a).

**Fig. 3:**
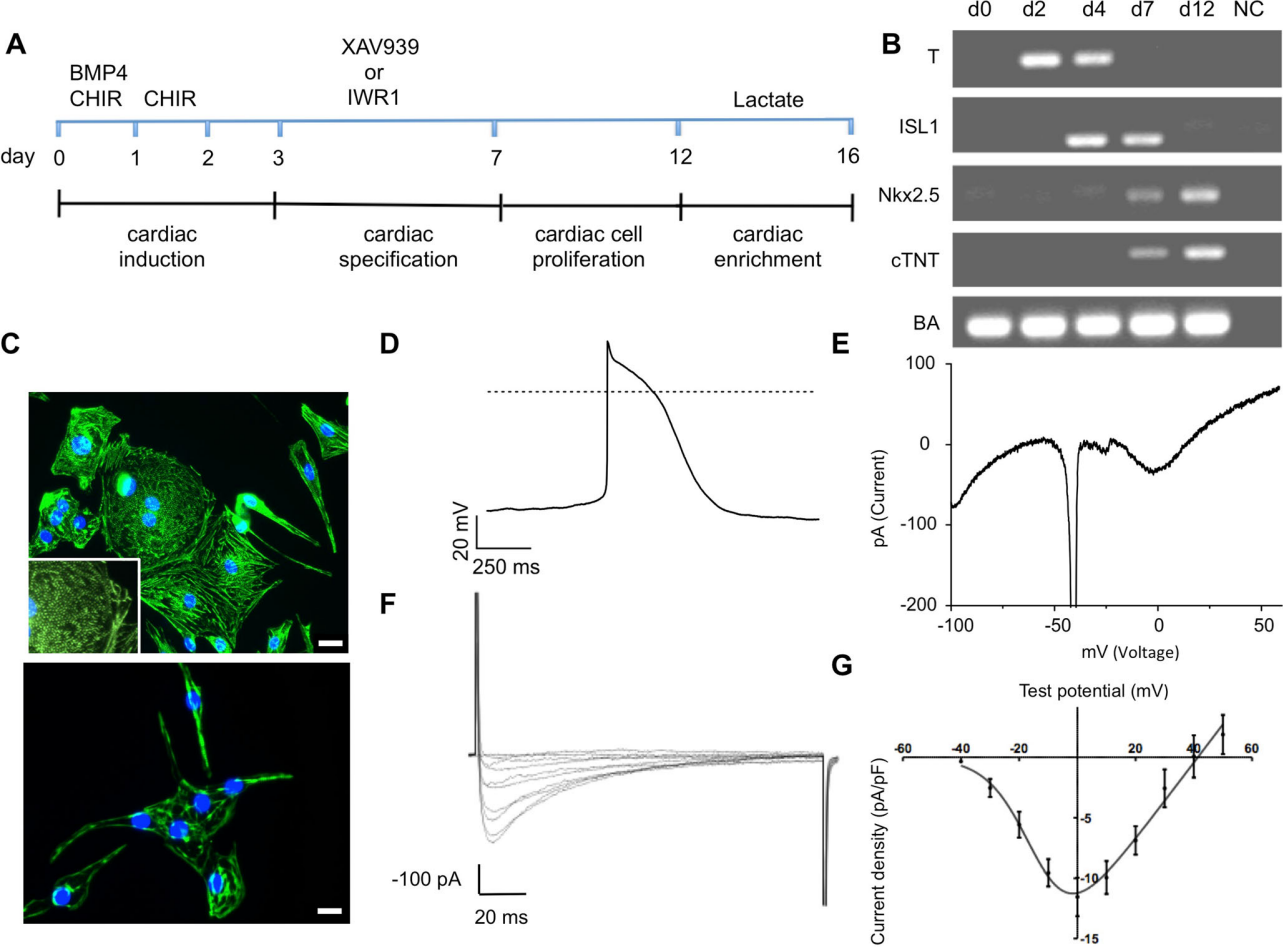
Characterization of human iPSC (del-AR1034ZIMA 001) derived cardiomyocytes. **a** Scheme of efficient cardiac differentiation of human iPSC with combination of strong cardiac induction in early phase and cardiac enrichment in late phase. **b** RT-PCR analysis for mesendoderm, mesoderm, and cardiac specific gene expression **c** Immunohistochemical characterization of hiPS-derived cardiomyocytes using antibody against alpha-actinin (top) and cardiac troponin T (bottom). Scale bar: 40 μm. **d** Action potential recorded from a ventricular like cardiomyocyte. **e** Typical activation of voltage dependent inward and outward currents following a ramp protocol in voltage clamp (-100 to +60 mV in 250 ms). **f** Representative whole cell calcium current recording (2 mM extracellular Ca^2+^). Cells were depolarized from a holding potential of -80 to -40 mV for 45 ms in order to inactivate sodium channels. This prepulse was followed by test voltages ranging from -40 to +50 mV in 10 mV steps (pulse duration 150 ms). (G) Whole cell calcium current density-voltage relationship (*n* = 4)

In conclusion our optimized protocol of cardiomyocyte differentiation from multiple human iPS lines represents a three phase protocol consisting of cardiac induction, specification and enrichment as outlined in Fig. 3a. During the induction phase iPS cells are treated with our formulation (BMP4 and CHIR) in a basal medium with insulin, which resulted in strong upregulation of the mesendodermal marker T-brachyury (Fig. 3b). Induction phase is followed by treatment with WNT inhibitors in basal medium devoid of insulin in order to achieve proper specification to cardiac mesoderm, which is confirmed by expression of early and late cardiac precursor markers ISL1 and Nkx2.5, respectively (Fig. 3b). Cells then further mature into beating cardiomyocytes expressing the mature cardiomyocyte marker cTNT (Fig. 3b). Once beating is observed cells are switched to basal medium with insulin followed by enrichment phase with basal medium devoid of glucose but supplemented with 4 mM lactate for 4 days (Fig. 3a).

### Characterization and Validation of Cardiomyocytes Derived from Human iPSCs

We performed a series of standard immunohistochemical and electrophysiological methods to assess the functionality of cardiomyocyte derived. Obtained cardiomyocytes showed strong cardiac specific alpha-actinin staining with typical striation pattern as well as cTNT staining (Fig. 3c). Moreover, patch-clamp recordings on single beating cardiomyocytes showed typical spontaneous action potentials (Fig. 3d), with atrial (*n* = 2) or ventricular (*n* = 4)-like properties, as well as characteristic voltage-dependent inward and outward currents using voltage ramps (Fig. 3e). We recorded Ca2+ currents typical for L-type Ca^2+^ channels with a half-maximum activation at −13.69 ± 0.97 mV, a maximum current density of −11.55 ± 1.6 pA/pF at 0 mV and nearly complete inactivation during 150 ms of depolarization (*n* = 4) (Fig. 3f and g). In addition calcium imaging was performed using the fluorescent Ca^2+^ indicator Fluo-4 AM. Fluo-4 AM fluorescence intensity sparks indicates binding of calcium to fluo-4 upon release of calcium from the sarcoplasmic reticulum in hiPS derived cardiomyocytes. Fluo-4 fluorescence intensity sparks were recorded using fluorescence microscopy (Supplementary movie S2).

### Ultra-Structural Analysis of iPS Cell-Derived Cardiomyocytes

**Fig. 4:**
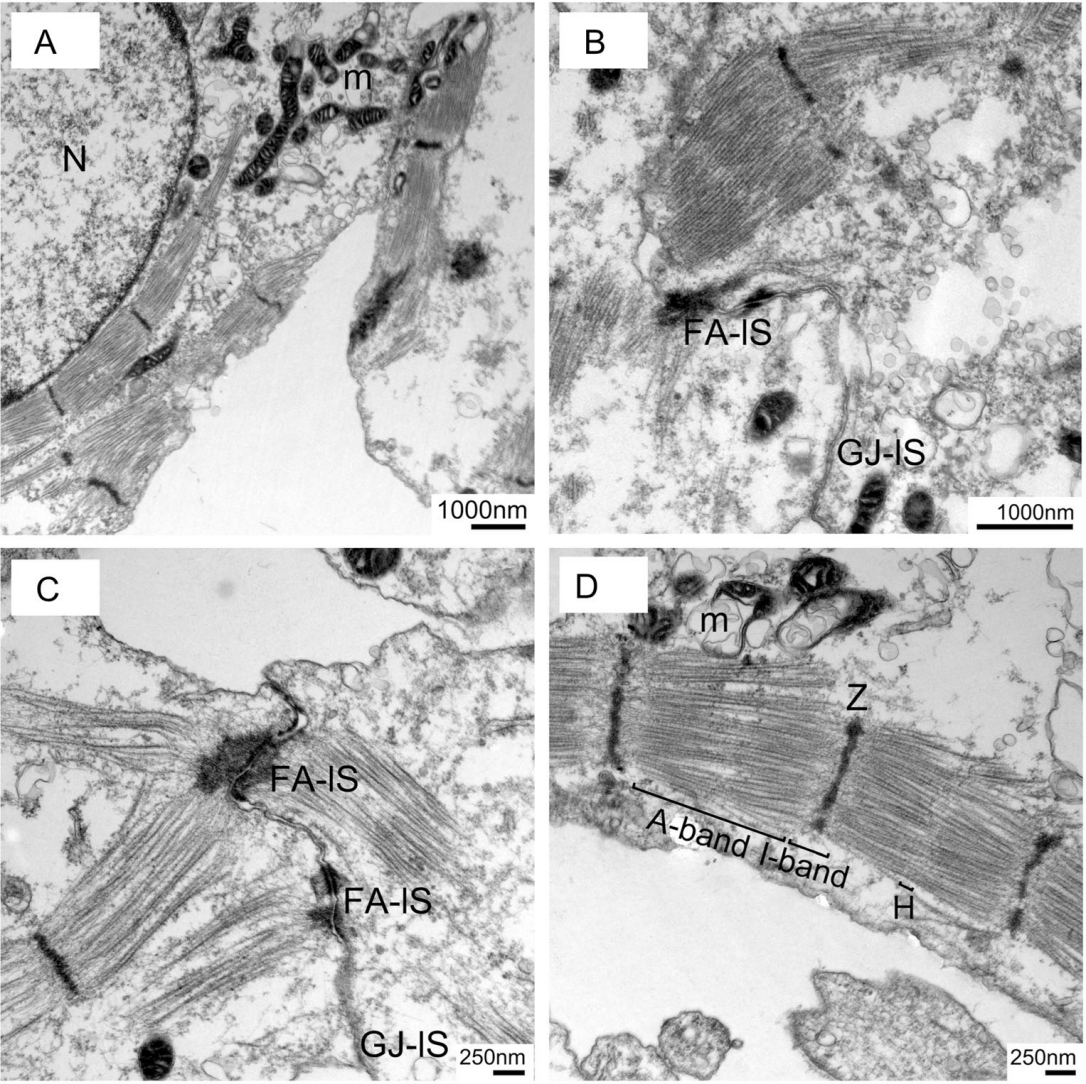
Ultrastructural analysis of 21-day old human iPS (del-AR1034ZIMA 001) cell-derived cardiomyocytes. **a** Two cells in close contact displaying sarcomer-like organization of contractile filaments. Scale bar: 1,000 nm (**b**–**c**) Higher magnification showing the presence of fascia adherens-like and gap-junctions like cellular contacts and initial sarcomeric organisation of actin and myosin filaments. Scale bar: **b** 1,000 nm, **c** 250 nm. **d** iPS cell-derived cardiomyocyte-like cells show sarcomer organization of contractile filaments with already identifiable A- and I-bands together with Z-lines as well as H-zones. Abbrevations: *m* mitochondria, *N* Nucleus, *FA-lS* Fascia adherens-like structure, *GJ-lS* Gap junctions-like structure, *Z* Z-line, *H* H-zone. Scale Bar: 250 nm

In order to study the maturation state of iPS-derived cardiomyocyte-like cells at ultra-structural level we performed transmission electron microscopy analysis of 21 day old cardiomyocytes. Many cells show nascent parallel arrays of myofilament bundles anchored at Z-line like electron dense structures (Suppl. Figure 1). They show different spatial orientation within the same cell as well as branching. Moreover, we observed sarcomer-like organization of contractile filaments (Fig. 4a). Additionally, fascia adherens-like and gap-junctions-like cellular contacts as well as initial sarcomeric organisation of actin and myosin filaments were detected. The sarcomeric structures of contractile filaments exhibited already identifiable A- and I-bands together with Z-lines as well as H-zones (Fig. 4b-d, Suppl. Figure 1).

## Discussion

Our study reports a novel robust strategy to obtain cardiomyocytes from diverse human iPS lines that originate from a wide spectrum of state-of-the-art reprogramming technology using an optimal combination of well-orchestrated extrinsic stimuli such as BMP4 and CHIR followed by WNT inhibition using XAV939 or IWR1 and enrichment of cardiomyocytes by supplying lactate as an energy source. The protocol described herein divides the whole differentiation process into three phases, namely cardiovascular induction, cardiac specification and cardiomyocyte enrichment. Our analysis revealed that efficient cardiac induction requires appropriate concentrations as well as precise timing of the right window of application of each chemical. We found out that sequential application of each chemical, BMP4 and CHIR, respectively, for up to two days is sufficient to drive the cells into cardiovascular fate which is consistent with earlier studies [28, 29, 31, 42]. Notably, we have used basal media with insulin during first two days of induction phase. Previous studies have reported use of basal medium without insulin during cardiac differentiation due to its negative influence on cardiac specification of early mesoderm [28, 39]. However, our study shows that using basal medium without insulin from the beginning appears very stressful to the cells. Hence we decided to keep insulin for the initial two days and remove it during the specification phase in order to minimize cell death and its negative influence on cardiac specification. In order to achieve cardiac specification of early mesoderm, we used inhibitors of WNT signaling for up to 4 days as described in earlier studies [28, 29]. We performed a comparative validation of molecules exhibiting WNT inhibitory activity using a WNT reporter cell line. It turned out that amongst four different compounds tested, XAV939 showed the strongest WNT inhibitory effect and was therefore used for all differentiation experiments. Moreover, we also achieved the same cardiac differentiation efficiency with previously described WNT inhibitor IWR-1 [27]. We observed different cardiac differentiation efficiencies in multiple iPS lines presumably due to the complexity of the signals, which is in accordance with recent studies [17, 18]. We therefore elaborated further purification steps to improve the yield of cardiomyocytes with reduced line-to-line variability. We decided to assess the potential of lactate enrichment of cardiomyocytes and show a substantial cardiac enrichment of an iPS line (iLB-C1-30 m-r12) that exhibited relatively poor cardiomyocyte yield using our optimized chemical cocktail only. This indicates that the combination of extrinsically induced differentiation stimuli together with metabolic enrichment is an efficient means to overcome line-to-line variability of cardiomyocyte differentiation. There are various published studies showing successful cardiac differentiation of human pluripotent stem cells. However, as depicted in supplementary table 4 three key features separates our protocol from others i.e. i) sequential treatment of cardiac inducing factor with a clearly defined time window together with ii) an insulin switch, which in our experience is very critical to get reproducible results; iii) in addition our protocol includes a rapid and efficient way to enrich the cardiac population with lactate supplement. In order to evaluate the functional properties of cardiomyocytes, comprehensive validation was not only carried out at protein level by alpha-actinin and cTNT stainings but also by ultra-structural analysis. TEM results showed well organized sarcomeric structures in 21 day old cardiomyocytes with distinct I- and A-bands indicating relatively mature phenotype as described in earlier studies [15, 29]. Spontaneous action potentials of differentiated cells showed typical cardiomyocyte behaviour and we identified both ventricular and atrial-like shapes. Voltage ramps identified fast sodium, calcium as well as potassium currents. Characteristics of Ca^2+^ currents obtained from iPS-derived cardiomyocytes were similar to those recently obtained from murine ventricular myocytes [40]. I-V relationship furthermore perfectly agreed with data from HEK293 cells expressing recombinant human L-type Ca^2+^-channels suggesting that indeed iPS-derived cells express cardiac-like channel complexes consisting of pore-forming and auxiliary subunits [41]. Moreover calcium imaging further indicated usual calcium current activity seen in the case of human cardiomyocytes. In conclusion, cardiomyocytes obtained by our protocol have the potential for stem cell based therapies, drug toxicity as well as disease modeling studies. Hence we expect our protocol to provide a robust basis for scale-up production of functional iPS cell-derived cardiomyocytes.

## Electronic Supplementary Material

Below is the link to the electronic supplementary material.

Supplementary Fig.1 Ultrastructural analysis of human iPS cells (del-AR1034ZIMA 001) 21 days after differentiation. (A-B) Representative transmission electron micrographs shows cells with rounded to elongated morphology forming fascia adhesion-like cellular contacts. Scale bar: 1000 nm. (C-D) Nascent parallel arrays of myofilament bundles anchored at Z-band like electron dense structures (D arrows). Scale bar: 1000 nm (E) Different spatial orientation of myofilament bundles within the same cell. Scale bar: 250 nm (F) Branching myofilament bundles. Scale Bar: 1000 nm. Abbreviations: FA-lS: Fascia adherens-like structure. (JPEG 110 kb)

Supplementary Table 1 Optimization of cardiac differentiation of human iPS line (iLB-C-50-s9) by varying concentration of CHIR in combination with 25 ng/ml of BMP4 and 10 μM IWR1. (DOCX 29 kb)

Supplementary Table 2 Optimization of cardiac differentiation of human iPS line (iLB-C-50-s9) by varying concentration of BMP4 in combination with 5 μM of CHIR and 10 μM IWR1. (DOCX 19 kb)

Supplementary Table 3 Optimization of cardiac differentiation of human iPS line (iLB-C-50-s9) by varying time window of WNT inhibition. (DOCX 20 kb)

Supplementary Table 4 Overview of selected recent studies showing successful cardiac differentiation of human iPS cells. (DOCX 24 kb)

Supplementary movie S1 Spontaneously beating cells at day 12 of cardiac differentiation of human iPS line (del-AR1034ZIMA 001) before lactate enrichment. (AVI 4263 kb)

Supplementary movie S2 Calcium imaging of cardiomyocytes obtained from human iPS cells (del-AR1034ZIMA 001) using the fluorescent Ca^2+^ indicator Fluo-4 AM. (MP4 94313 kb)
