# Supplementary figures and images for: Robust Generation of Cardiomyocytes from Human iPS Cells Requires Precise Modulation of BMP and WNT Signaling
Source: Stem Cell Rev. 2014 Nov 13;11(4):560–9. doi: 10.1007/s12015-014-9564-6 (PMC4493626; doi:10.1007/s12015-014-9564-6)

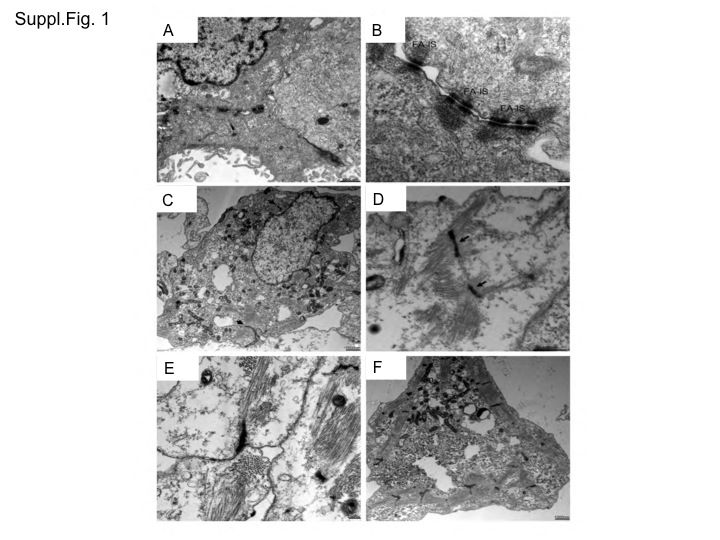

Supplement: Supplementary file 1 — Ultrastructural analysis of human iPS cells (del-AR1034ZIMA 001) 21 days after differentiation. (A-B) Representative transmission electron micrographs shows cells with rounded to elongated morphology forming fascia adhesion-like cellular contacts. Scale bar: 1000 nm. (C-D) Nascent parallel arrays of myofilament bundles anchored at Z-band like electron dense structures (D arrows). Scale bar: 1000 nm (E) Different spatial orientation of myofilament bundles within the same cell. Scale bar: 250 nm (F) Branching myofilament bundles. Scale Bar: 1000 nm. Abbreviations: FA-lS: Fascia adherens-like structure. (JPEG 110 kb) [file 12015_2014_9564_MOESM1_ESM.jpg]
