# Supplementary material for: Robust Generation of Cardiomyocytes from Human iPS Cells Requires Precise Modulation of BMP and WNT Signaling
Source: Stem Cell Rev. 2014 Nov 13;11(4):560–9. doi: 10.1007/s12015-014-9564-6 (PMC4493626; doi:10.1007/s12015-014-9564-6)
Supplement: Supplementary file 2 — Optimization of cardiac differentiation of human iPS line (iLB-C-50-s9) by varying concentration of CHIR in combination with 25 ng/ml of BMP4 and 10 μM IWR1. (DOCX 29 kb) [file 12015_2014_9564_MOESM2_ESM.docx]

| **Application of CHIR in combination with 24h BMP4 (25 ng/ml)** | | |
| --- | --- | --- |
| **Conc (μM). of CHIR** | **Time in hours** | **Extent of cardiac differentiation** |
| 5 | 24 | - |
| 5 | 48 | +++ |
| 5 | 72 | + |
| 10 | 24 | - |
| 10 | 48 | - |
| 10 | 72 | - |
| 15 | 24 | - |
| 15 | 48 | - |
| 15 | 72 | - |
| **Application CHIR in combination with 48h BMP4 (25 ng/ml)** | | |
| 5 | 24 | - |
| 5 | 48 | ++ |
| 5 | 72 | - |
| **Application of CHIR only** | | |
| 5 | 24 | - |
| 5 | 48 | + |
| 5 | 72 | - |
| 10 | 24 | + |
| 10 | 48 | - |
| 10 | 72 | - |
| 15 | 24 | - |

**Supplementary Table 1**

-, no beating; +, few beating patches; ++, numerous beating patches;

+++, synchronous beating throughout well
