# Supplementary material for: Robust Generation of Cardiomyocytes from Human iPS Cells Requires Precise Modulation of BMP and WNT Signaling
Source: Stem Cell Rev. 2014 Nov 13;11(4):560–9. doi: 10.1007/s12015-014-9564-6 (PMC4493626; doi:10.1007/s12015-014-9564-6)
Supplement: Supplementary file 3 — Optimization of cardiac differentiation of human iPS line (iLB-C-50-s9) by varying concentration of BMP4 in combination with 5 μM of CHIR and 10 μM IWR1. (DOCX 19 kb) [file 12015_2014_9564_MOESM3_ESM.docx]

**Supplementary Table 2**

| **Application of BMP4 in combination with 48h CHIR (5 μM)** | | |
| --- | --- | --- |
| **Conc (ng/ml). of BMP4** | **Time in hours** | **Extent of cardiac differentiation** |
| 12.5 | 24 | + |
| 25 | 24 | +++ |
| 50 | 24 | + |

+, few beating patches; +++, synchronous beating throughout well
