# Supplementary material for: Robust Generation of Cardiomyocytes from Human iPS Cells Requires Precise Modulation of BMP and WNT Signaling
Source: Stem Cell Rev. 2014 Nov 13;11(4):560–9. doi: 10.1007/s12015-014-9564-6 (PMC4493626; doi:10.1007/s12015-014-9564-6)
Supplement: Supplementary file 4 — Optimization of cardiac differentiation of human iPS line (iLB-C-50-s9) by varying time window of WNT inhibition. (DOCX 20 kb) [file 12015_2014_9564_MOESM4_ESM.docx]

**Supplementary Table 3**

| **Optimization of WNT inhibitor application** | |
| --- | --- |
| **Time window of application of 10 μM XAV939 or 10 μM IWR1** | **Extent of cardiac differentiation** |
| Day 1 to 3 | - |
| Day 2 to 5 | - |
| Day 3 to 6 | + |
| Day 3 to 8 | +++ |

-, no beating; +, few beating patches; +++, synchronous beating throughout well
