# Supplementary material for: Robust Generation of Cardiomyocytes from Human iPS Cells Requires Precise Modulation of BMP and WNT Signaling
Source: Stem Cell Rev. 2014 Nov 13;11(4):560–9. doi: 10.1007/s12015-014-9564-6 (PMC4493626; doi:10.1007/s12015-014-9564-6)
Supplement: Supplementary file 5 — Overview of selected recent studies showing successful cardiac differentiation of human iPS cells. (DOCX 24 kb) [file 12015_2014_9564_MOESM5_ESM.docx]

Suppl. Fig. 4

| **Cardiac induction factors** | **Cardiac specification factors** | **Cardiac enrichment**  **factors** | **Basal** **medium** | **Ref.** |
| --- | --- | --- | --- | --- |
| Activin A, BMP4 and bFGF | none | none | Stem Pro 34 (Insulin content not specified) | Carpenter et al., 2011 |
| CHIR | IWP4 | none | RPMI1640  (no-insulin) | Lian et al., 2013 |
| CHIR+BIO | KY02111 or  KY02111+XAV939 | none | IMDM  (Insulin content not specified) | Minami et al., 2013 |
| ActivinA, BMP4, CHIR | XAV939 | none | (LI)-BPEL  (Low-insulin) | Dambrot et al., 2014 |
| BMP4 , CHIR | XAV939 or IWR1 | Lactate | RPMI  (Insulin switch) | This study |
